# ﻿Mini-exon gene reveals circulation of TcI *Trypanosomacruzi* (Chagas, 1909) (Kinetoplastida, Trypanosomatidae) in bats and small mammals in an ecological reserve in southeastern Mexico

**DOI:** 10.3897/zookeys.1084.78664

**Published:** 2022-01-28

**Authors:** Eliza F. Gómez-Sánchez, Héctor Ochoa-Díaz-López, Eduardo E. Espinoza-Medinilla, D. Daniel Velázquez-Ramírez, Nancy G. Santos-Hernández, Christian Ruiz-Castillejos, Dolores G. Vidal-López, Adriana Moreno-Rodríguez, Any Laura Flores-Villegas, Eduardo López-Argueta, José A. De Fuentes-Vicente

**Affiliations:** 1 Laboratorio de Investigación y Diagnóstico Molecular, Instituto de Ciencias Biológicas, Universidad de Ciencias y Artes de Chiapas, Tuxtla Gutierrez, Mexico; 2 Departamento de Salud, El Colegio de la Frontera Sur, Tuxtla Gutierrez, Chiapas, Mexico; 3 Laboratorio Multidisciplinario Experimental y Bioterio, Instituto de Ciencias Biológicas, Universidad de Ciencias y Artes de Chiapas, Tuxtla Gutierrez, Mexico; 4 Laboratorio 16, Facultad de Ciencias Químicas, Universidad Autónoma Benito Juárez de Oaxaca, Oaxaca, Mexico; 5 Laboratorio de Biología de Parásitos, Facultad de Medicina, Universidad Nacional Autónoma de Mexico, Ciudad de México, Mexico

**Keywords:** Chagas disease, molecular epidemiology, reservoirs, sylvatic cycle

## Abstract

A wide variety of mammals are involved in the sylvatic cycle of *Trypanosomacruzi*, the causative agent of Chagas disease. In many areas in Latin America where *T.cruzi* is endemic, this cycle is poorly known, and its main reservoirs have not been identified. In this study we analyzed *T.cruzi* infection in bats and other small mammals from an Ecological Reserve in southeastern Mexico. From January through March 2021, we captured wild individuals to extract cardiac and peripheral blood, and infection was detected by PCR of the mini-exon gene. In bats, the prevalence of infection was 16.36%, while in small mammals the prevalence was 28.57%. All of the samples that were positive for *T.cruzi* were identified as the TCI genotype. Our findings suggest that this zone, situated at the periphery of urban zones might have epidemiological relevance in the sylvatic cycle of *T.cruzi* and needs to be monitored. The infection of bats in this area is particularly concerning since the flight pattern of this populations overlaps with human settlements. Despite being subject to conservation protections, there continue to be anthropogenic actions that disturb the study area, which could exacerbate risks to public health.

## ﻿Introduction

The protozoan *Trypanosomacruzi* (Chagas, 1909) (Kinetoplastida, Trypanosomatidae) is the causative agent of Chagas disease, a neglected tropical infection affecting ~6 million people (Krats 2019). This disease typically occurs in rural areas of Central and South America, but urban areas are not exempt. In humans, chronic *T.cruzi* infection leads to heart failure and death in 20–30% of infected patients (WHO 2014; Krats 2019). Chagas disease is difficult to diagnosis, and only two drugs, Nifurtimox and Benznidazole, are currently available to treat it, both of which have severe side effects (Vallejo et al. 2020).

*Trypanosomacruzi* exhibits high genetic variability and has recently been classified into six discrete typing units (DTUs; TCI–TCVI) and an additional unit named TC Bat (see Zingales et al. 2018). Under natural conditions, *T.cruzi* is transmitted by blood-sucking insects of the subfamily Triatominae (Hemiptera, Reduviidae) known as kissing bugs (De Fuentes-Vicente and Gutiérrez-Cabrera 2020). *Trypanosomacruzi* transmission cycles are well defined into domestic, peridomestic, and sylvatic cycles, each with epidemiological and ecological differences. The domestic and peridomestic cycles involve humans, pets (dogs and cats), and farmyard animals. In sylvatic cycles in wild habitats, marsupials, edentates, and rodents are important reservoirs, but *T.cruzi* can infect more than 100 different species of wild mammals (Noireau et al. 2009; Alvarado-Otegui et al. 2012). This heterogeneity suggests a highly variable ecology of *T.cruzi*, and each area may have a unique set of conditions underlying the occurrence of the parasite (Moreira-Alves et al. 2016).

Historically, the domestic and peridomestic cycles have been the most studied, and little is known about the sylvatic cycle, especially in the southeastern region of Mexico (e.g., Jimenez-Coello et al. 2012). The climatic and biodiversity conditions of this region, in addition to poverty and marginalization, create scenarios for increased occurrence of Chagas disease (see Cruz-Reyes and Pickering-López 2006). In the current study, we sought to determine the infection by *T.cruzi* in wild mammals from the “El Zapotal” Ecological Reserve using multiplex PCR amplification of the mini-exon gene. “El Zapotal” is located in the state of Chiapas in southeastern Mexico, and we believe that this area may have epidemiological importance in the sylvatic cycle of *T.cruzi* in the region and that the proximity of human settlements may make it relevant to public health. In fact, the circulation of *T.cruzi* in small mammals in this area has previously been reported (Domínguez-Vázquez et al. 1990; Solís-Franco et al. 1997; Camacho-Sierra 2016). In addition, this area has high bat species richness, including synanthropic species (Velazquez-Pérez et al. 2010; López-Argueta 2021). Although bats have played a key role in the evolution of *T.cruzi* (Hamilton et al. 2012), their importance in the transmission dynamics has been poorly studied in many regions.

Although “El Zapotal” is subject to conservation protections, anthropogenic actions may have already caused irreversible damage (Fernández-Moreno 2010). Large-scale changes in land use and habitat fragmentation can affect wild transmission cycles of *T.cruzi* (Vaz et al. 2007), mostly because habitat loss restricts the area and food resources available to wild mammals, which can increase their contact with humans. All these factors support the need to conduct new studies to better understand the dynamics of *T.cruzi* transmission in wild ecotopes.

## ﻿Methods

### ﻿Study site

**Figure 1.**
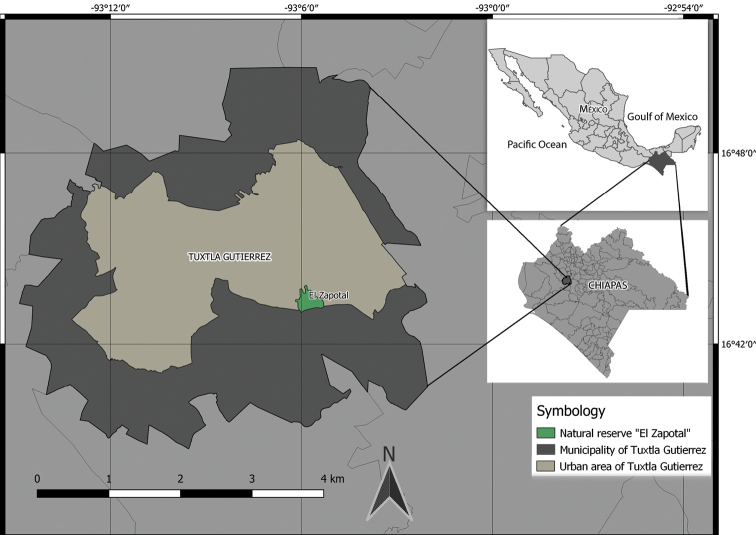
Location of the “El Zapotal” Ecological Reserve in southeastern Mexico, note the border of the reserve with the urban area.

The “El Zapotal” Ecological Reserve, decreed as an Ecological and Recreational Park, is located 2 km southeast of Tuxtla Gutiérrez, Chiapas (Fig. 1). It is a natural protected area measuring approximately 200 ha. The geology is largely karstic, with abundant caves and springs. The altitudinal range is from 600 to 850 m above sea level and the vegetation is medium sub-evergreen forest and low deciduous forest.

### ﻿Mammal capture and blood sampling

Wild mammals were captured from January through March 2021 in areas of the “El Zapotal” Ecological Reserve near bodies of water and fruit trees. To capture bats, we deployed three 12 × 2.5 m mist nets from dusk to dawn (eight sampling hours per net) for five consecutive nights. The captured specimens were deposited in canvas bags for identification and blood sampling. The identification was performed as described by Díaz (2021). Meanwhile, the blood sample was obtained by intracardiac puncture (100 μL) and deposited in microcentrifuge tubes with 500 μL 3.8% sodium citrate pH 7.2 for their transportation to the laboratory. Finally, the bats were marked on the wings with ink and released on site.

For the capture of smalls mammals 20 Tomahawk type traps and 15 Sherman traps were used (Romero-Almaraz et al. 2000). As bait, a mixture of oats with vanilla extract was used. The traps were set at dusk and removed eight hours later for five nights. Captured individuals were marked and identified as described by Reid (2009), and a blood sample was taken by puncture in the tail vein, after disinfecting the area with 70% alcohol. The samples obtained (100 μL) were treated as mentioned above.

### ﻿Bioethical guidelines

Animal handling was carried out in accordance with the provisions of Mexican Animal Welfare Law. The capture of animals was approved by the Mexican Secretariat of the Environment and Natural Resources (Secretaría de Medio Ambiente y Recursos Naturales, SEMARNAT (minute 07 / K6-0095 / 10/189)). No individuals were sacrificed or removed from the site.

### ﻿Extraction of DNA and mini-exon gene amplification

Total DNA was extracted using a modified phenol-chloroform isoamyl alcohol protocol (Espinoza and García 2003). For the amplification of the mini-exon gene, we used a pool of three oligonucleotides reported by Souto et al. (1996): [5'-GTGTCCGCCACCTCCTTCGGGCC (TCI, group 1-specific), 5'-CCTGCAGGCACACGTGTGTGTG (TCII, group 2-specific), and 5'-CCCCCCTCCCAGGCCAC ACTG (TC, common to groups TCI and TCI)]. We used the previously characterized strains Querétaro (TCI) and strain Y (TCII), which amplify at 350 and 300 base pairs (bp), respectively, as controls (Espinoza et al. 2010). Amplification reactions were performed in a final volume of 25 μL, containing 12 μL of Go Taq Green Master Mix 2X, 10 μL of nuclease-free water, 0.4 μM of each primer, and 20 ng of Trypanosoma DNA. Cycle amplification was performed using a MyGene MG96G thermal cycler (Hangzhou LongGene Scientific Instruments Co. Ltd, Hangzhou, China) under the following conditions: 5 min at 94 °C, followed by 27 cycles of 40 s at 94 °C, 40 s at 61 °C, and 1 min at 72 °C, and a final elongation of 5 min at 72 °C. Amplified products were visualized on 2% W/V agarose gels stained with ethidium bromide under UV light.

## ﻿Results

A total of 152 mammals were captured: 110 bats and 42 small mammals. Among bats, eight species were identified, and *Artibeusjamaicensis* Leach, 1821 (Chiroptera, Phyllostomidae) was the most common species. Only two hematophagous individuals (*Desmodusrotundus* É. Geoffroy, 1810) (Phyllostomidae) were captured. We captured four species of small mammals, of which *Didelphismarsupialis* Linnaeus, 1758 (Didelphimorphia, Didelphidae) was the most common (Table 1).

**Table 1.** Bats and smalls mammals captured in “El Zapotal” ecological reserve and infected individuals.

Bats			
Family	Species	# individuals	Infected indivuals (% prevalence)
Phyllostomidae	*Artibeusjamaicensis*	64	10 (15.6)
	*Artibeuslituratus*	16	3 (18.7)
	*Sturniralillium*	3	2 (66.6)
	*Centuriosenex*	2	0
	*Leptonycterisyebabuenae*	2	0
	*Carolliaperspicillata*	7	2 (28.5)
	*Desmodusrotundus*	2	0
	*Glossophagasoricina*	8	1 (12.5)
	*Pteronotusdavyi*	1	0
Mormoopidae	*Pteronotusparnelli*	3	0
	*Mormoopsmegallophyla*	2	0
**Total**		**110**	**18 (16.3)**
**Small mammals**			
Didelphidae	*Didelphismarsupialis*	18	6 (33.3)
Cricetidae	*Peromyscusmexicanus*	7	4 (57.1)
Heteromyidae	*Heteromysdesmarestianus*	10	1 (10)
Dasyproctidae	*Dasyproctamexicana*	7	1 (14.2)
**Total**		**42**	**12 (28.5)**

**Figure 2.**
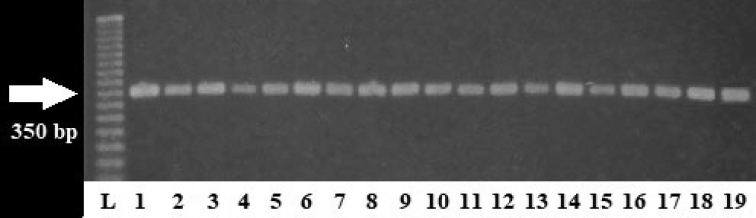
PCR products of the mini-exon gene in blood of bats from the “El Zapotal” Ecological Reserve. Amplification resulted in a PCR product of 350 bp and this confirms that these parasites belong to the TCI group. L: Ladder; Samples: 1 positive control (Qro. strain); 2–11 *A.jamaicensis*; 12–14 *A.lituratus*; 15–16 *C.perspicillata*; 17–18 *S.lilium*; 19 *G.soricina*.

Of the total bat samples examined, 18 were positive for *T.cruzi* infection (16.36%). *Sturniralilium* É. Geoffroy, 1810 had the highest prevalence among the bat species (66.6%), though only three individuals were captured. Meanwhile, the most commonly captured bat species, *A.jamaicensis*, had a prevalence of 15.62% (10/64) (Table 1). All PCR products amplified at 350 bp, indicating that they belonged to the TCI group (Fig. 2).

**Figure 3.**
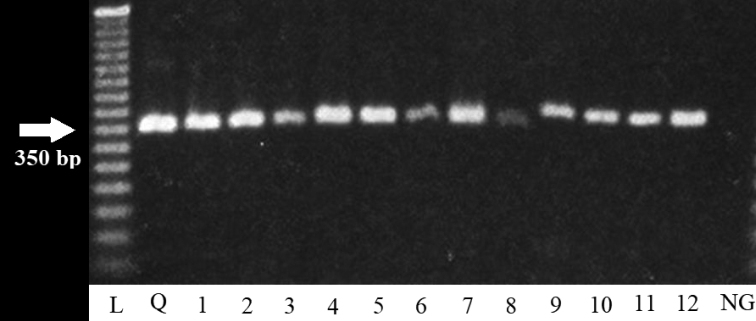
PCR products of the mini-exon gene in blood of smalls mammals from the “El Zapotal” Ecological Reserve. Amplification resulted in a PCR product of 350 bp and this confirms that these parasites belong to the TCI group. L: ladder; samples: Q positive control (Qro. strain); 1–6 *D.marsupialis*; 7–10 *P.mexicanus*; 11 *H.desmarestianus*; 12 *D.mexicana*.

For smalls mammals there was an overall prevalence of 28.57% (12/42), when combining all four mammal species. *Peromyscusmexicanus* (Saussure, 1860) (Rodentia, Cricetidae) presented the highest prevalence with 57.14% (4/7), while the most commonly captured species, *D.marsupialis*, had a prevalence of 33.33% (6/18) (Table 1). Here too, all PCR products amplified at 350 bp indicating the TCI group of *T.cruzi* (Fig. 3).

## ﻿Discussion

We show evidence of the circulation of *T.cruzi* in wild mammals from an ecological reserve in southeastern Mexico. Although other studies have demonstrated the presence of the parasite in small mammals from “El Zapotal” (Domínguez-Vázquez et al. 1990; Solís-Franco et al. 1997; Camacho-Sierra 2016), the present study is the first to report infection in bats. TCI was the only genetic group detected. This genetic group is the most prevalent in Mexico (Bosseno et al. 2002; Dorn et al. 2017) and is associated with *Triatomadimidiata* Latreille, 1811 (Hemiptera, Triatominae), the main *T.cruzi* vector in Central and North America (López-Cancino et al. 2015). Some *T.cruzi* genotypes have close evolutionary relationships with specific triatomine species, possibly favoring parasite transmission (De Fuentes-Vicente et al. 2019).

Overall, the prevalence of *T.cruzi* infection was 19.73% in all captured individuals (30/152). The overall infection prevalence in small mammals (28.57%) was similar to previous findings in a recent study (26.66%) in the same area (Camacho-Sierra 2016) and higher than in bats. We found a higher prevalence of infection in *P.mexicanus* (Mexican mouse) than in *D.marsupialis* (common opossum). The fact that small rodents are an important food source for several predators could maintain the transmission of *T.cruzi* among mammals through predation. In addition, vertical or congenital transmission has been demonstrated in these animals (Alarcón et al. 2009). Other studies in southern Mexico have also reported high prevalence of *T.cruzi* circulation in terrestrial mammals (e.g., Ruiz-Piña and Cruz-Reyes 2002; Martínez-Hernández et al. 2014), including in livestock (sheep, pigs, and horses) and urban and rural dogs in Yucatán (Jiménez-Coello et al. 2008; Ruiz-Piña et al. 2018).

To date, it is largely unknown how the sylvatic cycle interacts with the peridomestic and domestic cycles, but it is inferred that some synanthropic animals may be the link between them. For example, some synanthropic rodents captured in Yucatán have shown histological lesions associated with *T.cruzi* infection (Torres-Castro et al. 2016; Ucan-Euan et al. 2019). “El Zapotal” is surrounded by urbanized human settlements, and infected rodents might represent a public health risk due to their ability to invade and colonize human dwellings, where they could interact with domestic animals and parasite transmission could occur. For example, in the neighboring city of Tuxtla Gutiérrez, a prevalence of 4.5% of *T.cruzi* infection in stray dogs has been reported (Jiménez-Coello et al. 2010), and recently the first report of an infected triatomine bug in the urban area was published (De Fuentes-Vicente et al. 2020).

In Mexico, the dynamics of *T.cruzi* in bats in the sylvatic cycle has been little studied, even though bats have wide distributions that may overlap with urbanized environments (Krauel and Lee Buhn 2016), as occurs in the populations analyzed here (López-Argueta 2022). The synanthropic condition of bats has made them the transmitters of several pathogens including Ebola virus, rabies, and hantaviruses (Calisher et al. 2006; Kasso and Balakrishnan 2013). Currently, they are the focus of increased attention because of their possible relationship with the origin of the novel SARS-COV-2 coronavirus that causes COVID-19 (Lau et al. 2020; Córdoba-Aguilar et al. 2021). In particular, bats play a role of interest in the evolution of *T.cruzi* because, according to some hypotheses, *T.cruzi* evolved from a larger clade of bat trypanosomes (Hamilton et al. 2012). The importance of bats as reservoirs of *T.cruzi* may be enhanced by their ability to fly, gregarious social structure, and longevity (Luis et al. 2013). In bats, we found a prevalence of infection of 16.36%, a value much higher than that found in the only previous study in Chiapas, which sampled bats from the Selva Lacandona (1.60%) (Víquez-Rodríguez 2015). Interestingly, they reported a higher prevalence of infection by *Leishmaniamexicana* in the same individuals (8.84%) and only one bat infected by both (Víquez-Rodríguez 2015). Although bats are known to be associated with a wide range of zoonotic pathogens, the effects of competition between parasites in the same reservoir remains virtually unknown (Bashey 2015).

High prevalence of *T.cruzi* infection in bats have been previously reported in southern Mexico: Torres-Castro et al. (2021) reported a 30.2% prevalence of infection in bats from Campeche and Yucatan, mostly in non-hematophagous species. We only collected two hematophagous individuals (*D.rotundus*), neither of which was infected. Non-hematophagous species may acquire the parasite by ingesting infected insects or by vector transmission, but we did not find any triatomine insects at the study sites. Vertical transmission of *T.cruzi* has also been demonstrated in bats (Añez et al. 2009), so this mechanism may also favor the permanence of the parasite in these animals. Another interesting fact in this group is that *T.cruzi* was detected in the salivary glands of a hematophagous bat specimen in Peru (Villena et al. 2018), suggesting that the importance of bats in the dynamics of *T.cruzi* may be greater than previously thought, since they may be able to transmit the parasite directly through biting.

Maintaining biodiversity has been shown to be an important – if not the most important – action to prevent the spread of zoonotic parasites (Córdoba-Aguilar et al. 2021), and *T.cruzi* is no exception (Keesing and Ostfeld 2021). As such, we must continue to explore how ecosystem fragmentation affects sylvatic transmission cycles of *T.cruzi*, a topic which is further complicated by heterogeneity in the reservoirs, vectors, and genetic structure of the parasites. Further studies of all of these topics are necessary in order to construct effective interventions that prevent the sylvatic cycle from connecting with the peridomestic or domestic cycle and further exposing humans. Finally, future research should emphasize the role of bats in the dynamics of *T.cruzi* to determine their role in the epidemiology of Chagas disease and to inform health authorities about this potential danger.
